# Spontaneous Eruption of Permanent Teeth That Had Eruption Disturbances After Extirpation of Odontomas: A Report of Two Cases

**DOI:** 10.7759/cureus.61460

**Published:** 2024-05-31

**Authors:** Yuki Kunisada, Norie Yoshioka, Kyoichi Obata, Koki Umemori, Soichiro Ibaragi

**Affiliations:** 1 Department of Oral and Maxillofacial Surgery, Okayama University Graduate School of Medicine, Okayama, JPN

**Keywords:** orthodontic treatment, spontaneous eruption, tooth eruption, permanent tooth, odontoma

## Abstract

Odontomas, often found adjacent to impacted teeth, are tumors of abnormal tissue morphology arising from the tooth germ and are usually asymptomatic. They are often found by accident on X-ray images, and the eruption of permanent teeth is often caused by odontomas. In most cases, the tooth is extracted with the permanent tooth or orthodontic treatment is performed after extraction. However, the criteria are not clear. We encountered two cases of dental eruption in which permanent teeth, which originally seemed to be suitable for orthodontic treatment, spontaneously erupted after odontoma removal. It is necessary to examine the indications and timing of tooth extraction.

## Introduction

Odontoma is the most common odontogenic tumor classified as a benign mixed epithelial and mesenchymal odontogenic tumor by the World Health Organization (WHO) in 2017 [1]. Most odontomas are asymptomatic, and they are often discovered by accident on X-ray imaging [2]. In some cases, permanent tooth eruption defects may be caused by odontomas [3-6]. The treatment strategy for an embedded tooth whose eruption is impaired by odontoma depends on the condition of the tooth [7,8]. Generally, impacted teeth with eruption disturbances caused by odontomas are indicated for extraction if the impacted tooth has complete roots, strong bone adhesion, a significantly inclined tooth axis, or a reverse impacted tooth [9]. In recent years, since the development of orthodontic treatment, there are often cases of teeth being tractioned by orthodontic treatment after surgery [10]. If it is not in the lower position and the tooth axis direction is relatively good, conservative therapy is often selected with the expectation of eventual spontaneous eruption [11,12]. However, the criteria are not clear, and tooth extraction is often accepted. Here, we report the summary of two cases where impacted teeth, which would likely be indicated for orthodontic treatment or extraction along with odontoma removal, unexpectedly spontaneously erupted after odontoma extraction and erupted into the correct position.

## Case presentation

Case 1

**Figure 1:**
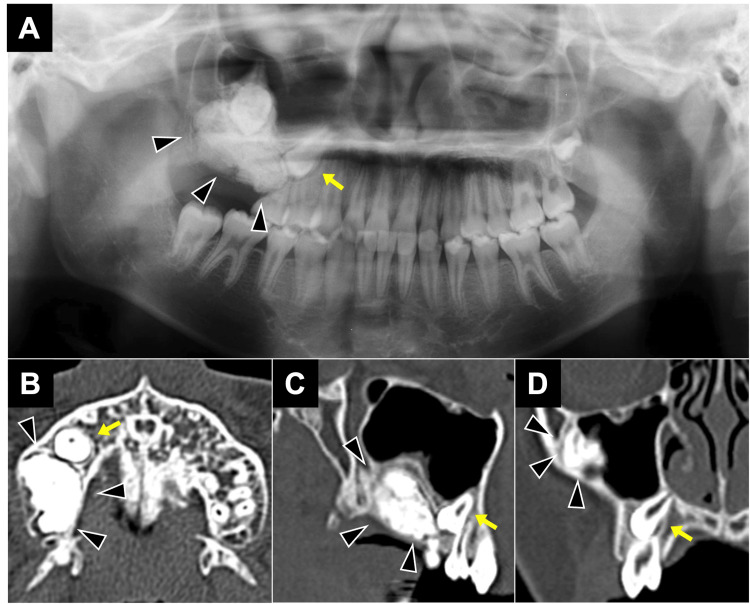
CBCT images at the first examination - Case 1. (A) Panoramic radiograph depicting well-defined mass in the right upper molar region. (B-D) Opaque images with clear boundaries measuring 35×25×15 mm were observed in axial (B), sagittal (C), and coronal (D) views, and the maxillary right second premolar was deviated as arrow. Arrowheads indicate odontoma. CBCT: cone-beam computed tomography

A 12-year-old male patient was referred to our department from his regular dental clinic for further examination and treatment because of a lesion in the right upper molar region (Figure 1A) and the prolonged retention of a deciduous tooth of the deciduous second molar of the right upper jaw, and maxillary right first and second premolar eruption delay. On computed tomography (CT) imaging, a clear-marginated radiopaque lesion was observed at his right upper molar region, and a diagnosis of compound odontoma was made (Figures 1B-1D). The lesion caused the floor of the maxillary sinus to be elevated, the posterior permanent teeth to be deviated upwards, and the maxillary right lateral second premolar to be deviated medially upwards. The impacted tooth had incomplete root formation, no shrinkage or enlargement of the tooth follicle, and no abnormal tooth morphology. Surgical removal of the odontoma was performed under general anesthesia. The occlusal surface of the impacted tooth was directly visible from the wound site, and it was confirmed that the tooth was swaying, and the operation was completed as an open wound.

**Figure 2:**
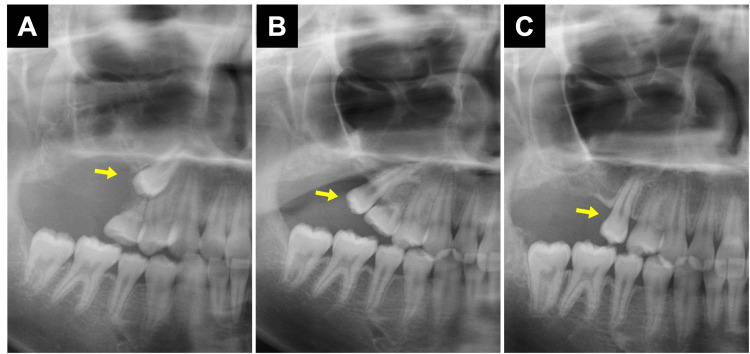
The condition of tooth after surgery. The images show the condition of tooth (A) zero months after the operation, (B) 12 months after the operation, and (C) 21 months after the operation. Each arrow indicates an impacted tooth.

After surgery, the maxillary right second premolar naturally erupted distally to the maxillary right second deciduous molar. This tooth was extracted 17 months later, but the maxillary right second premolar moved naturally to the correct position without orthodontic treatment (Figures 2A-2C).

Case 2

**Figure 3:**
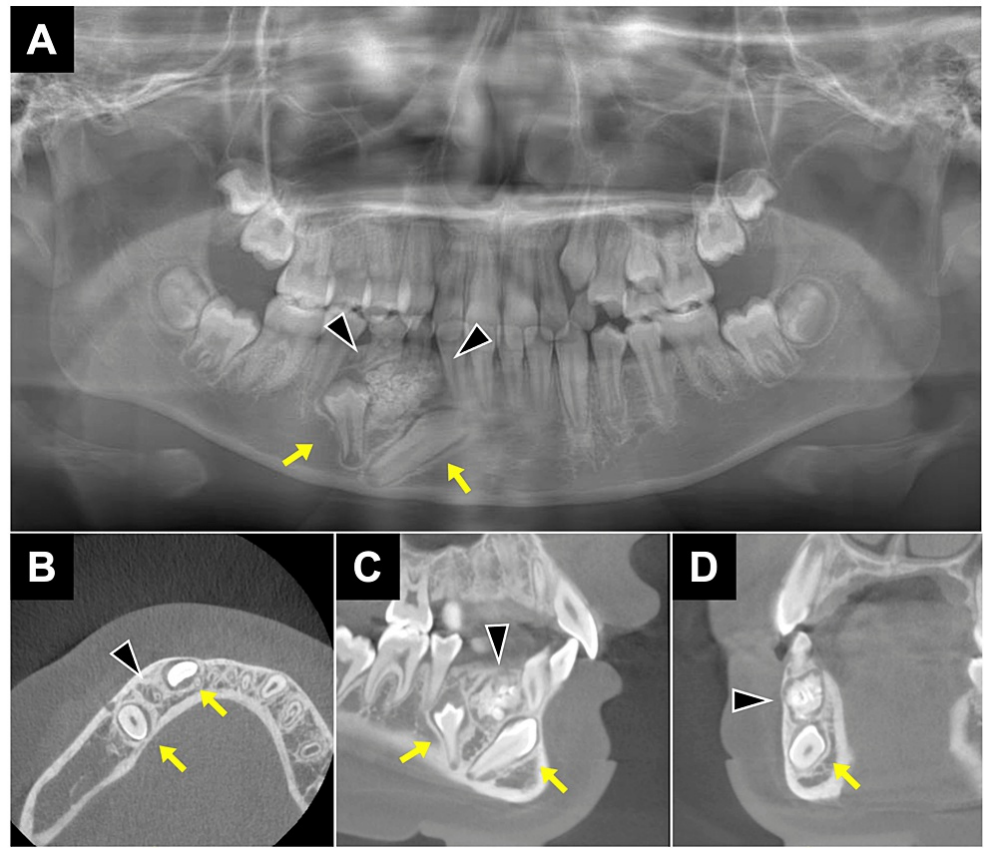
CBCT images at the first examination - Case 2. (A) Panoramic radiograph depicting well-defined mass in the mandible premolar region. (B-D) Opaque images with clear boundaries were observed in axial (B), sagittal (C), and coronal (D) views, and the canine and second premolar were deviated as arrow. Arrowheads indicate odontoma. CBCT: cone-beam computed tomography

A 10-year-old female patient was referred to our department for further examination and treatment at her regular dental clinic because of the prolonged retention of a deciduous tooth of the mandibular deciduous molar and canine and second premolar in the right lower jaw eruption delay. On CT imaging, a clear-marginated radiopaque lesion was observed in the right mandibular premolar region, and a diagnosis of complex odontoma was made (Figures 3A-3D). The odontoma was situated at the root apex of the deciduous canine and first deciduous molar, and between the canine and first premolar. Due to the lesion, the canine moved comparatively anteriorly and inferiorly, while the first premolar moved slightly outward and downward. In the sagittal and frontal sections, the odontoma and the canine were partly close to each other, but not in contact. The canine crown was located on the buccal side of the apex of the right lower lateral incisor, but was intact. The impacted tooth had an incomplete root, no shrinkage or enlargement of the tooth follicle, and no abnormal tooth morphology. Extraction of the odontoma was performed under general anesthesia.

**Figure 4:**
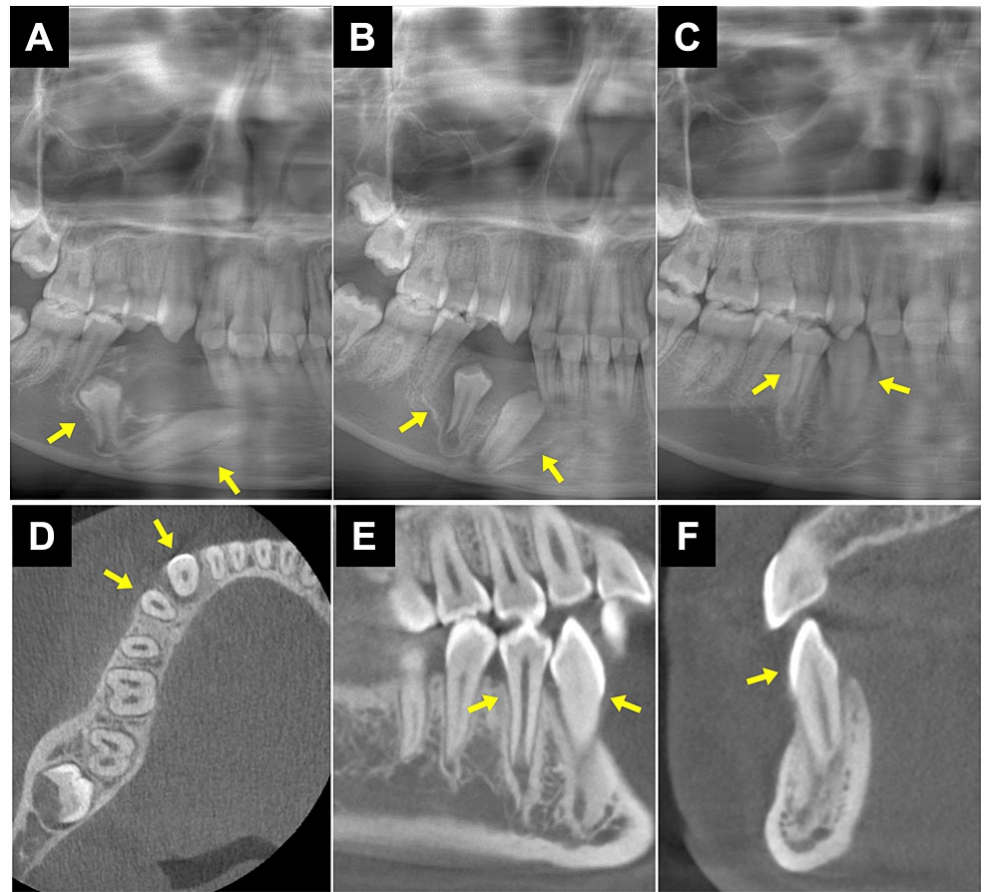
The condition of the tooth after surgery and postsurgical panoramic reconstructed CBCT image. The images show the condition of tooth (A) zero months after the operation, (B) three months after the operation, and (C) 15 months after the operation. (D) Axial, (E) sagittal, and (F) coronal views of CBCT taken 15 months after surgery. Each arrow indicates an impacted tooth, and the arrow in (F) indicates the right mandibular canine. CBCT: cone-beam computed tomography

We were able to see directly the crown tip of the impacted tooth from the wound, and it was confirmed that the tooth did not adhere to the bone, and the operation was completed as an open wound. The right mandibular canine spontaneously improved the tooth axis three months after surgery, and one year after surgery, we confirmed that the right mandibular canine had erupted normally (Figures 4A-4C). At that time, cone-beam computed tomography (CBCT) imaging was performed, and as shown in Figures 4D-4F, the right mandibular canine and the right mandibular first premolar were found to have erupted naturally into their normal positions.

## Discussion

The treatment of an impacted tooth depends on whether it erupts after extraction of the odontoma. However, the criteria to determine whether or not the teeth spontaneously erupt are not clear, and if orthodontic treatment is not indicated, the tooth is often extracted. Even when orthodontic treatment is indicated, there are cases in which it is not possible due to economic difficulties. In addition, the patient may have a strong vomiting reflex, which makes it difficult to make a model for orthodontic treatment, or the orthodontic treatment itself may be difficult because of the inability to insert the appliance into the mouth. Therefore, it seems that tooth extraction is selected in most cases. It has been reported that about 75% of impacted teeth that are considered caused by eruption disorders due to odontoma can be expected to spontaneously erupt after extraction [13]. Ashkenazi et al. analyzed the spontaneous eruption rate of impacted teeth associated with odontoma was lower than for teeth associated with supernumerary. Further, they reported spontaneous eruption after surgery (including supernumerary) correlated with the apex distance of the impacted tooth relative to its estimated position, extent of vertical impaction, obstacle form, stage of root development of the supernumerary tooth, angle of impaction relative to the midline, and time of surgery [3].

In Case 1, the patient was told that the second premolar was deviated more proximally downward than its original position due to odontoma and that natural eruption might be difficult. We explained that if the tooth did not erupt spontaneously, another extraction surgery would be necessary, but at the patient's request, we decided to preserve the tooth for follow-up observation. In Case 2, the tooth was inclined due to odontoma, especially since the canine was close to the root apex of the lateral incisor. We explained to the patient that spontaneous eruption might be difficult because of the strong inclination of the tooth axis and the proximal direction of eruption. Therefore, we suggested extraction or orthodontic traction. The patient preferred orthodontic traction, so we performed conservative treatment. However, the patient had a strong vomiting reflex and was difficult to impress, so we decided to wait and see the condition without treatment. Contrary to our expectations, however, in both cases, the teeth moved to the correct position after the operation. We hypothesized that natural eruption of impacted teeth could be induced if certain conditions were met, including incomplete roots, retention of tooth follicles, absence of morphological abnormalities, no contact with surrounding teeth, sufficient space for eruption, confirmable mobility during operation, confirmable crown tip, sufficient bone cutting, and a secure guide route. Preoperative X-ray images show incomplete roots, retention of tooth follicles is essential, and in addition, no morphological abnormalities of the teeth are observed, there is no contact with surrounding teeth, and there is space for tooth eruption. Additionally, it is necessary to be able to confirm the mobility of the impacted tooth during the operation, to be able to confirm the tip of the crown from the tumor excision site, to perform sufficient bone cutting, and to secure the guide route. However, since only two cases have been studied, it is difficult to draw definite conclusions, and further accumulation of cases is necessary.

## Conclusions

It is expected that the use of orthodontic treatment will shorten the treatment period and increase certainty. If orthodontic treatment is not an option, many cases are likely to result in tooth extraction, however, it may be possible to induce natural eruption by creating an environment that facilitates tooth eruption. When a guide route is formed in an impacted tooth, there is a risk of infection due to its deep location making cleaning difficult. However, postoperative gauze packing and proper cleaning can reduce this risk. These risks should be explained, including the risk of leaving the teeth and the potential for secondary surgery. These options should be considered if informed consent is obtained. Further follow-up must be performed carefully and regularly.
